# Supplementary material for: Identification of a HTT-specific binding motif in DNAJB1 essential for suppression and disaggregation of HTT
Source: Nat Commun. 2022 Aug 10;13:4692. doi: 10.1038/s41467-022-32370-5 (PMC9365803; doi:10.1038/s41467-022-32370-5)
Supplement: Supplementary file 1 — Supplementary Information [file 41467_2022_32370_MOESM1_ESM.pdf]

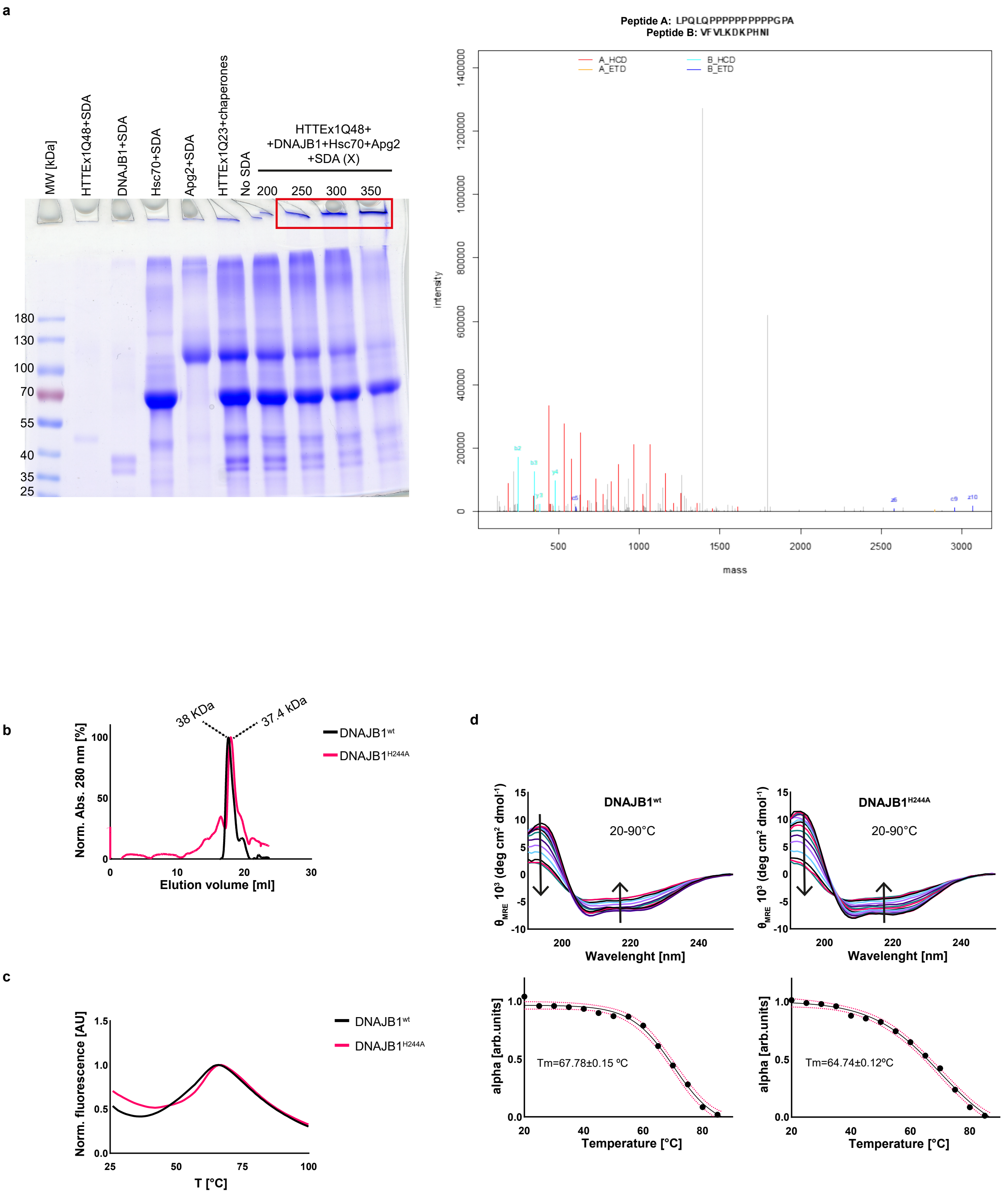
a
MW [kDa]
HTTEx1Q48+SDA
DNAJB1+SDA
Hsc70+SDA
Apg2+SDA
HTTEx1Q23+chaperones
No SDA
HTTEx1Q48+
+DNAJB1+Hsc70+Apg2
+SDA (X)
200 250 300 350
180
130
100
70
55
40
35
25
Peptide A: LPQLQPPPPPPPPPPGPA
Peptide B: VFVLKDKPHNI
A_HCD
A_ETD
B_HCD
B_ETD
intensity
mass
b
Norm. Abs. 280 nm [%]
Elution volume [ml]
38 KDa
37.4 kDa
DNAJB1wt
DNAJB1H244A
c
Norm. fluorescence [AU]
T [°C]
DNAJB1wt
DNAJB1H244A
d
DNAJB1wt
20-90°C
DNAJB1H244A
20-90°C
θMRE 10³ (deg cm² dmol⁻¹)
Wavelenght [nm]
alpha [arb.units]
Temperature [°C]
Tm=67.78±0.15 ºC
Tm=64.74±0.12ºC

**Supplementary figure 1. XL-MS analysis and integrity analysis of DNAJB1 variants a)** Left, representative SDS–PAGE of cross-linked proteins. The gel piece highlighted in red box was excised and in-gel digested for LC-MS analysis. We have performed more than ten independent crosslinking analyses of HTTExon1$Q_n$ and the chaperones. The depicted gel is a representative example. Right, the MS2 spectrum of an identified cross-link. **b)** Size exclusion chromatography of DNAJB1$^{wt}$ and DNAJB1$^{H244A}$. **c)** Melting curve of DNAJB1$^{wt}$ (red) and DNAJB1$^{H244A}$ (black) obtained by differential scanning fluorescence using SYPROrange dye. **d)** Top, CD spectra of DNAJB1$^{wt}$ and DNAJB1$^{H244A}$ obtained over a temperature range of 20-90°C. Bottom, calculation of the melting temperature (Tm), which is defined as $\alpha$ = 0.5.

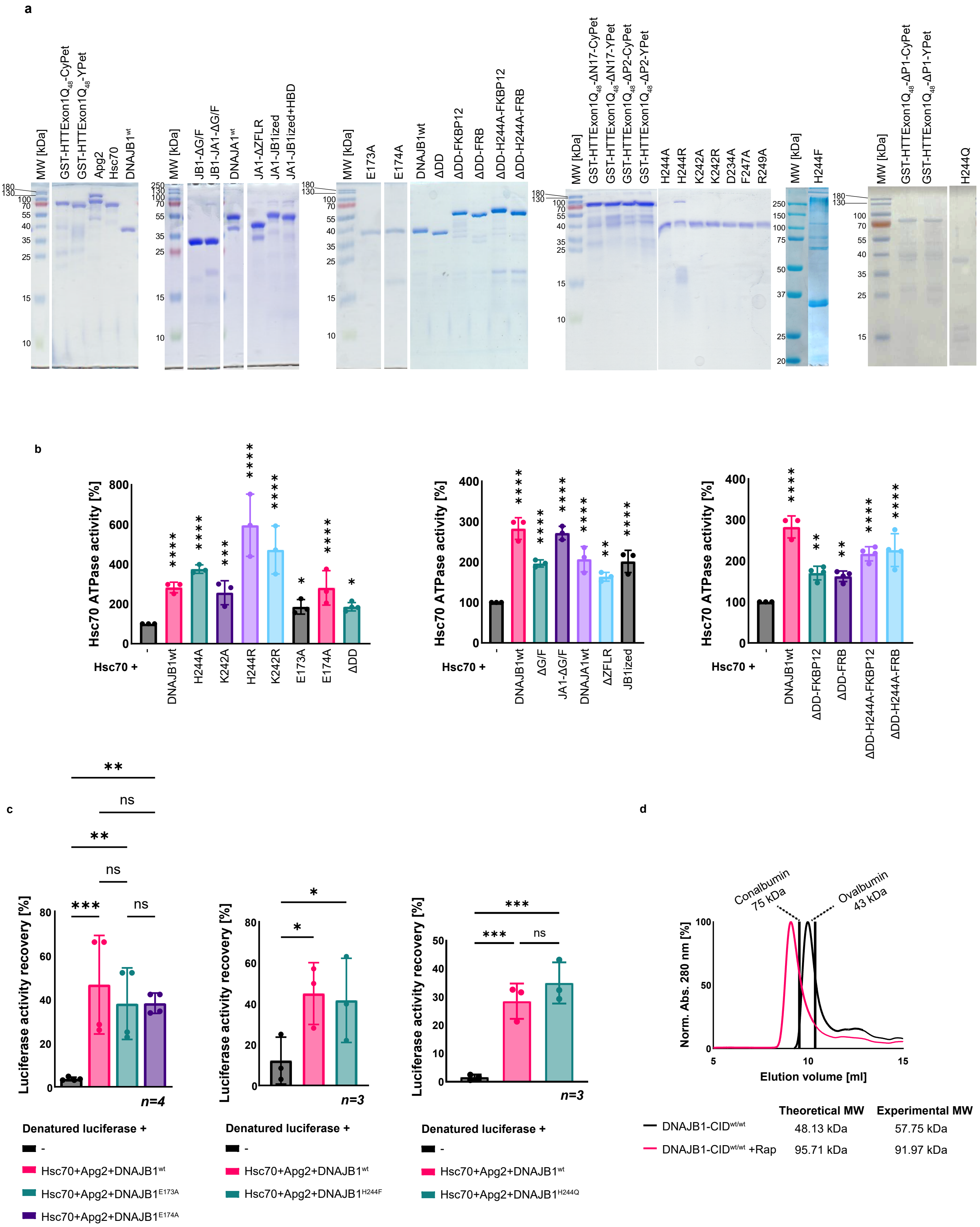
a
MW [kDa]
GST-HTTExon1Q48-CyPet
GST-HTTExon1Q48-YPet
Apg2
Hsc70
DNAJB1wt
JB1-ΔG/F
JB1-JA1-ΔG/F
DNAJA1wt
JA1-ΔZFLR
JA1-JB1ized
JA1-JB1ized+HBD
E173A
E174A
DNAJB1wt
ΔDD
ΔDD-FKBP12
ΔDD-FRB
ΔDD-H244A-FKBP12
ΔDD-H244A-FRB
GST-HTTExon1Q48-ΔN17-CyPet
GST-HTTExon1Q48-ΔN17-YPet
GST-HTTExon1Q48-ΔP2-CyPet
GST-HTTExon1Q48-ΔP2-YPet
H244A
H244R
K242A
K242R
D234A
F247A
R249A
H244F
GST-HTTExon1Q48-ΔP1-CyPet
GST-HTTExon1Q48-ΔP1-YPet
H244Q
b
Hsc70 ATPase activity [%]
Hsc70 +
DNAJB1wt
H244A
K242A
H244R
K242R
E173A
E174A
ΔDD
ΔG/F
JA1-ΔG/F
DNAJA1wt
ΔZFLR
JB1ized
ΔDD-FKBP12
ΔDD-FRB
ΔDD-H244A-FKBP12
ΔDD-H244A-FRB
c
Luciferase activity recovery [%]
n=4
n=3
n=3
Denatured luciferase +
-
Hsc70+Apg2+DNAJB1wt
Hsc70+Apg2+DNAJB1E173A
Hsc70+Apg2+DNAJB1E174A
Hsc70+Apg2+DNAJB1H244F
Hsc70+Apg2+DNAJB1H244Q
d
Conalbumin 75 kDa
Ovalbumin 43 kDa
Norm. Abs. 280 nm [%]
Elution volume [ml]
Theoretical MW
Experimental MW
DNAJB1-CIDwt/wt 48.13 kDa 57.75 kDa
DNAJB1-CIDwt/wt +Rap 95.71 kDa 91.97 kDa

**Supplementary figure 2. Analysis of purified proteins and control chaperone assays a)** SDS-PAGE analysis of all purified proteins used in this study. At least three batches of each protein were independently overexpressed and purified. 15 µl of 2 µM of each protein were subjected to SDS-PAGE with subsequent Coomassie staining. **b)** ATPase activity of Hsc70 alone and upon addition of DNAJB1$^{wt}$ and variants. Three independent experiments were performed and all data were normalized to the intrinsic ATPase activity of Hsc70. One-way ANOVA analysis was used to evaluate the significance of the difference observed between Hsc70 alone and Hsc70 plus a DNAJB1 variant. The extend of ATPase activation of Hsc70 by the different DNAJB1 variants could be due to altered affinities to Hsc70. Bars represent the mean value and error bars correspond to the mean SD. ****, $p \leq 0{,}0001$; ***, $p \leq 0{,}001$; **, $p \leq 0{,}01$; *, $p \leq 0{,}1$. **c)** Recovered enzymatic activity of luciferase after 2 h incubation with Hsc70, Apg2 and DNAJB1$^{wt}$ or variants E173A, E174A (left), H244F (middle), and H244Q (right). Data from three independent experiments were analyzed with a one-way ANOVA test. Bars represent the mean value and error bars correspond to the mean SD. ***, $p \leq 0{,}001$; **, $p \leq 0{,}01$; *, $p \leq 0{,}1$; *ns*, not significant. **d)** Size exclusion chromatography of DNAJB1$^{\Delta DD}$-FKBP12 and DNAJB1$^{\Delta DD}$-FRB in the absence (black) or presence (magenta) of Rapamycin. The elution volumes of MW standards Conalbumin and Ovalbumin are indicated on top and were used to calculate the experimental MW of the proteins.

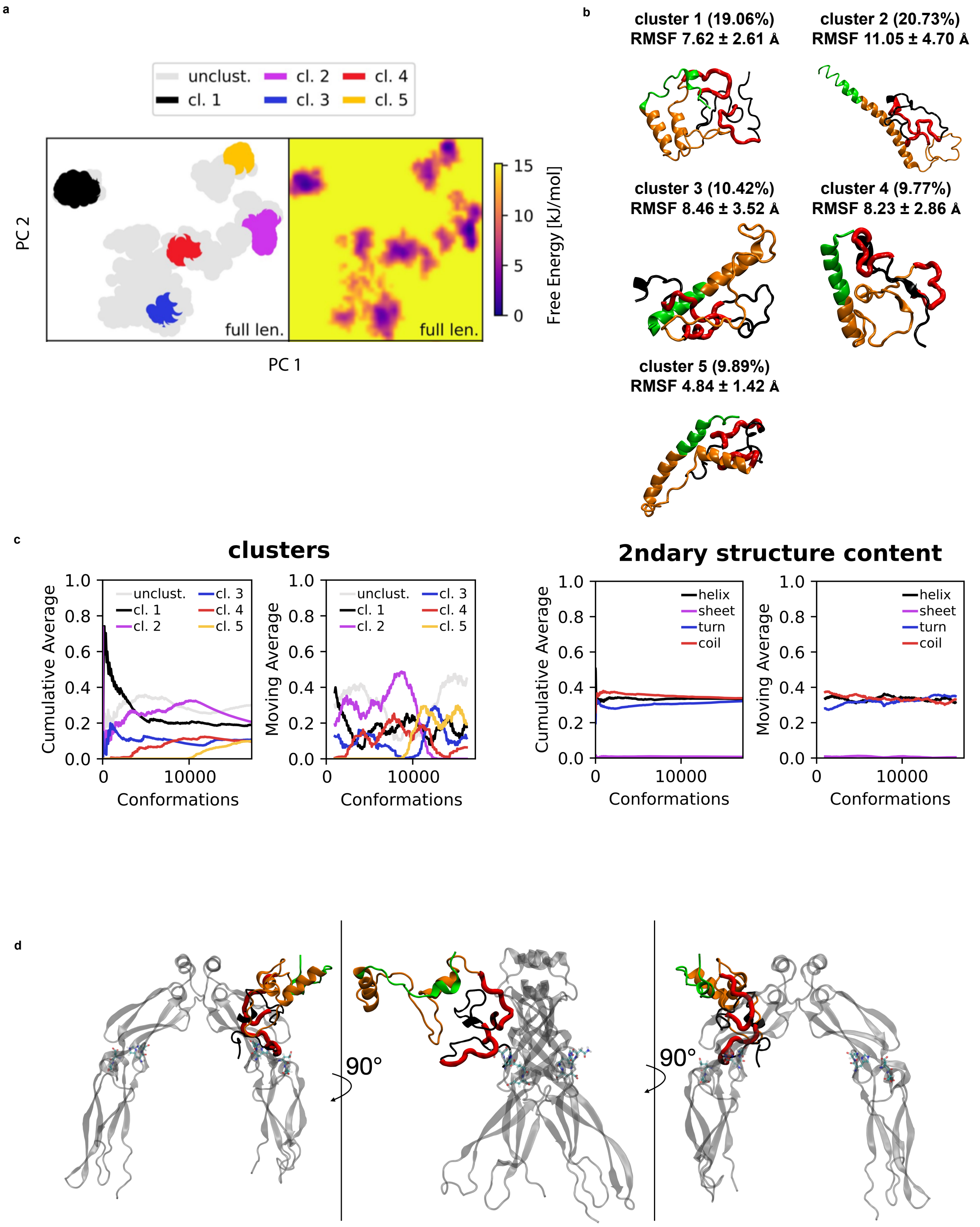
a
unclust.
cl. 1
cl. 2
cl. 3
cl. 4
cl. 5
PC 2
PC 1
full len.
full len.
15
10
5
0
Free Energy [kJ/mol]
b
cluster 1 (19.06%)
RMSF 7.62 ± 2.61 Å
cluster 2 (20.73%)
RMSF 11.05 ± 4.70 Å
cluster 3 (10.42%)
RMSF 8.46 ± 3.52 Å
cluster 4 (9.77%)
RMSF 8.23 ± 2.86 Å
cluster 5 (9.89%)
RMSF 4.84 ± 1.42 Å
c
clusters
2ndary structure content
Cumulative Average
Moving Average
Conformations
0
10000
0.0
0.2
0.4
0.6
0.8
1.0
helix
sheet
turn
coil
d
90°
90°

**Supplementary figure 3. Structure prediction of HTTExon1Q$_{48}$ and docking to DNAJB1 a)** Dihedral principal component analysis (dPCA) of HTTExon1Q$_{48}$ with the corresponding density-based clustering analysis. The colors indicate the respective clusters (left) and the free energy in kJ/mol (right). **b)** Respective structural clusters determined by dPCA analysis with their probability and root mean square fluctuation (RMSF). The average RMSF values are measured within these clusters comparing the protein backbone atoms to the averaged positions after aligning all conformations to the first structure. The N17 domain is depicted in green, the polyQ stretch in orange, the P1 and P2 of the PRD in red and the residues between P1 and P2 in black. **c**) Cumulative and moving average fractions for the most populated clusters (left) and the secondary structure content (right). Conformations are ordered after the occurrence time in the base line replica at 310 K. The moving average window size was set to 2000 conformations. The helical content in cluster 1 and cluster 3 converged fast during the replica exchange simulation, while other clusters 2, 4 and 5 converged slower and might have not reached the equilibrium probability within the simulation time. **d)** Equilibrated complex of DNAJB1 and HTTExon1Q$_{48}$ after 320 ns of classical MD simulation. Rotation around the symmetry axis of DNAJB1 reflects binding of HTTExon1Q$_{48}$ on one protomer only. The binding site on the second protomer is kept free.

a

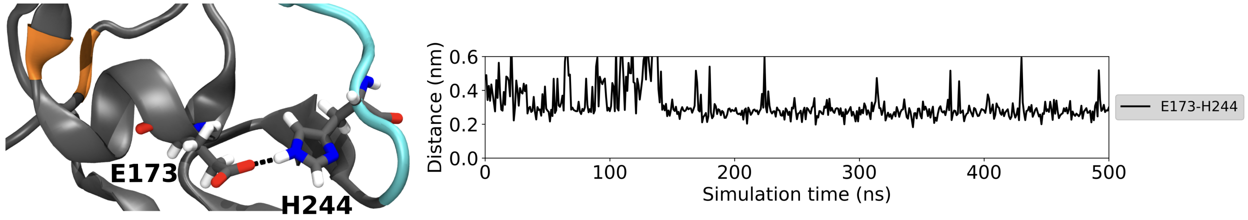
E173
H244
Distance (nm)
0.0
0.2
0.4
0.6
0
100
200
300
400
500
Simulation time (ns)
E173-H244

b

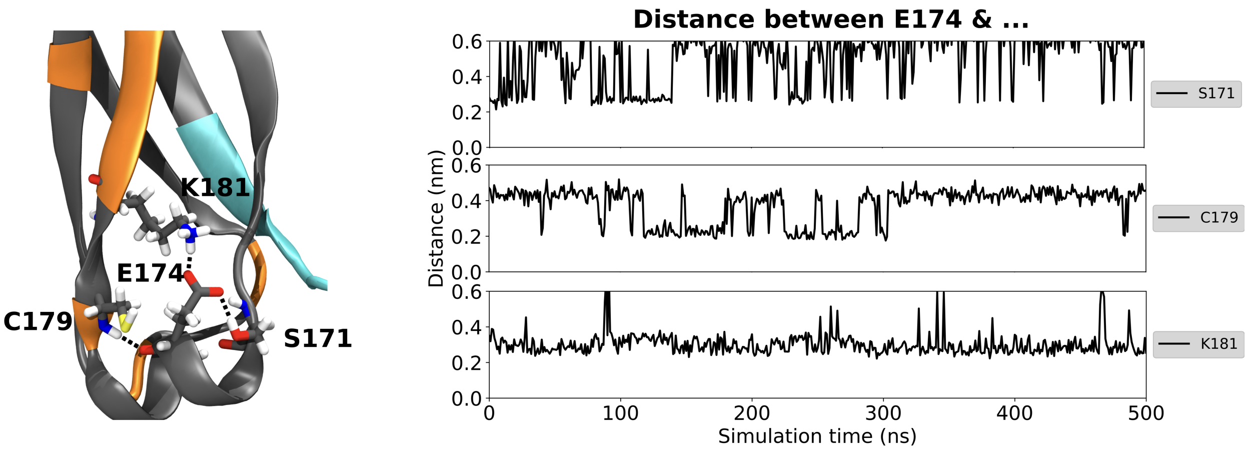
K181
E174
C179
S171
Distance between E174 & ...
Distance (nm)
S171
C179
K181
0.0
0.2
0.4
0.6
0
100
200
300
400
500
Simulation time (ns)

**Supplementary figure 4. MD snapshot of the hinge region between CTDI and CTDII a)** Visualization of the hydrogen bond (black dotted line) between E173 and H244A. The distance between hydrogen bond forming atoms of E173 (center of both oxygens) and H244 were recorded for DNAJB1$^{wt}$ over the simulation time of 500 ns (right panel). **b)** Analogous, the hydrogen bonds between E174 and S171, C179 and K181 are highlighted with black dashed lines in the molecular snapshot and the time evolution over 500 ns plotted. DNAJB1 is represented in cartoon style in gray. Highlighted amino acids of DNAJB1 are represented as licorice style with a coloring code according to the atom types: hydrogen (white), carbon (cyan), oxygen (red), nitrogen (blue). The HBM is colored in cyan.

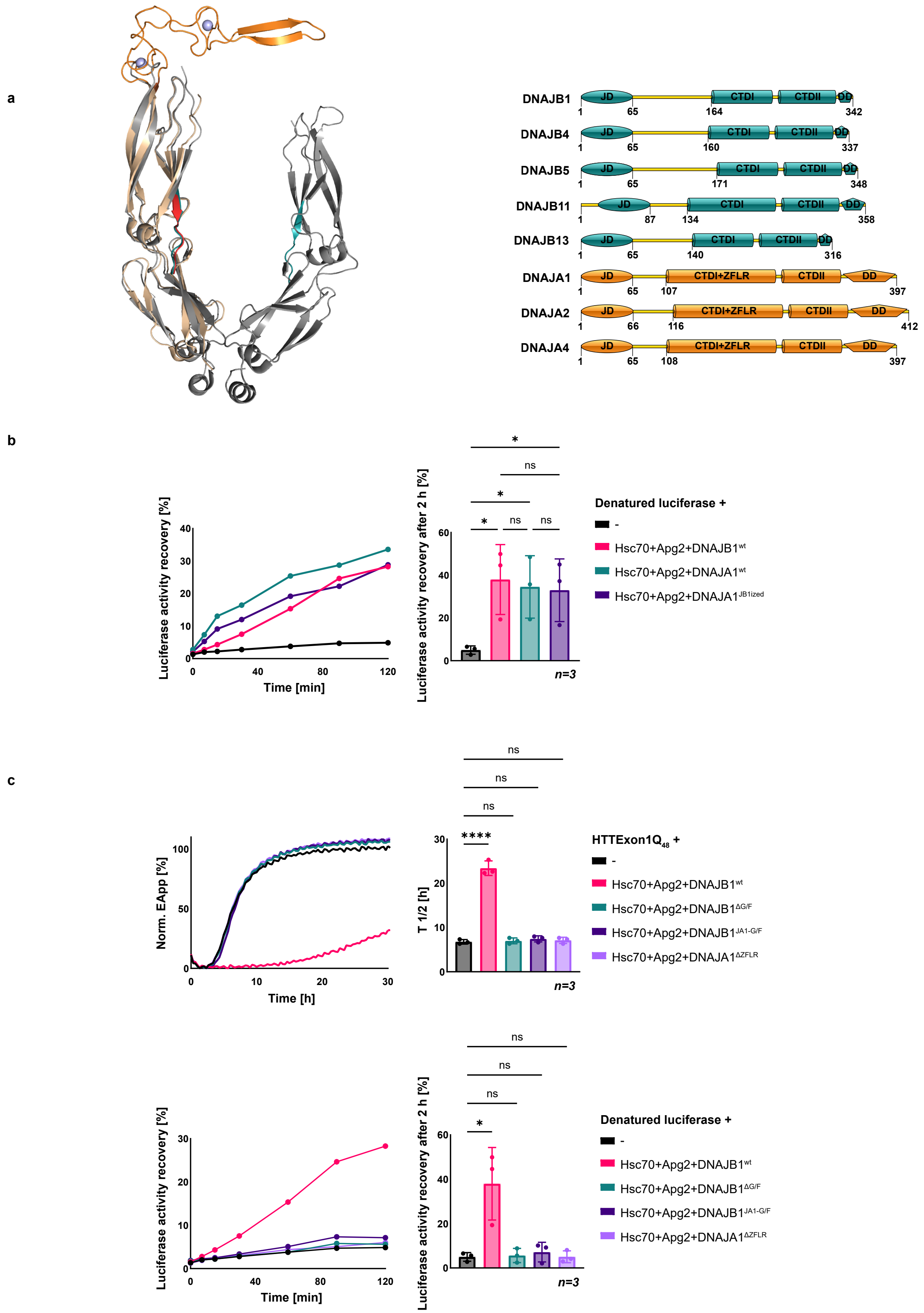
a
DNAJB1
JD
CTDI
CTDII
DD
1
65
164
342
DNAJB4
1
65
160
337
DNAJB5
1
65
171
348
DNAJB11
1
87
134
358
DNAJB13
1
65
140
316
DNAJA1
CTDI+ZFLR
1
65
107
397
DNAJA2
1
66
116
412
DNAJA4
1
65
108
397
b
Luciferase activity recovery [%]
Time [min]
Luciferase activity recovery after 2 h [%]
*
ns
n=3
Denatured luciferase +
-
Hsc70+Apg2+DNAJB1wt
Hsc70+Apg2+DNAJA1wt
Hsc70+Apg2+DNAJA1JB1ized
c
Norm. EApp [%]
Time [h]
T 1/2 [h]
****
HTTExon1Q48 +
-
Hsc70+Apg2+DNAJB1wt
Hsc70+Apg2+DNAJB1ΔG/F
Hsc70+Apg2+DNAJB1JA1-G/F
Hsc70+Apg2+DNAJA1ΔZFLR
Luciferase activity recovery [%]
Time [min]
Luciferase activity recovery after 2 h [%]
Denatured luciferase +
-
Hsc70+Apg2+DNAJB1wt
Hsc70+Apg2+DNAJB1ΔG/F
Hsc70+Apg2+DNAJB1JA1-G/F
Hsc70+Apg2+DNAJA1ΔZFLR

**Supplementary figure 5. Domain architecture of different JDPs and analysis of DNAJB1 variants in chaperone assays a)** Left, structural overlay of a dimer of CTDs of DNAJB1$^{wt}$ (in gray) and a monomer of CTD of DNAJA1$^{wt}$ (in sand). The C-terminal domain model of DNAJA1 was build based on the highly homologous CTD of yeast Ydj1 (pdb: 1nlt) using the Prime homology modeling tool of the Maestro Software Suite Version 12.7 (Schrödinger LLC). The most striking difference between the two structures is the protrusion of the ZFLR from the CTDI of DNAJA1$^{wt}$ (in orange). The remaining structures of DNAJB1$^{wt}$ and DNAJA1$^{wt}$ CTDs, including the HBM (green and red β-sheets), overlap. Right, schematic alignment of human J-domain proteins from class A (orange) and class B (teal). The G/F-rich domains (depicted in yellow and connecting the J-domains with the CTDs) of class B J-domain proteins are significantly longer than those of class A J-domain proteins. **b)** Refolding of denatured luciferase by Hsc70, Apg2 and DNAJB1$^{wt}$, DNAJA1$^{wt}$ or DNAJA1$^{JB1ized}$. The graph is a representative result of three independent experiments. A one-way ANOVA analysis of the amount of refolded luciferase after 2 h of incubation with chaperones is depicted on the right. Bars represent the mean value and error bars correspond to the mean SD. **, $p$ < 0,5; *ns*, not significant. **c)** Effect of Hsc70, Apg2 and DNAJB1$^{wt}$, DNAJB1$^{\Delta G/F}$ (deletion of G/F-rich domain), DNAJB1$^{JA1\text{-}G/F}$ (G/F-rich domain substituted by the shorter G/F-rich domain of DNAJA1), DNAJA1$^{wt}$ or DNAJA1$^{\Delta ZFLR}$ (ZFLR deleted) on HTTExon1$Q_{48}$ aggregation (top) and refolding of heat-denatured luciferase (bottom). The graphs on the left are representative results of three independent experiments. The bar graphs on the right are the one-way ANOVA analysis of the half-life ($T_{1/2}$) of HTTExon1$Q_{48}$ aggregation (top) and the amount of refolded luciferase after 2 h (bottom). Error bars correspond to the SD. ****, $p$ ≤ 0,0001; **, $p$ < 0,5; *ns*, not significant.

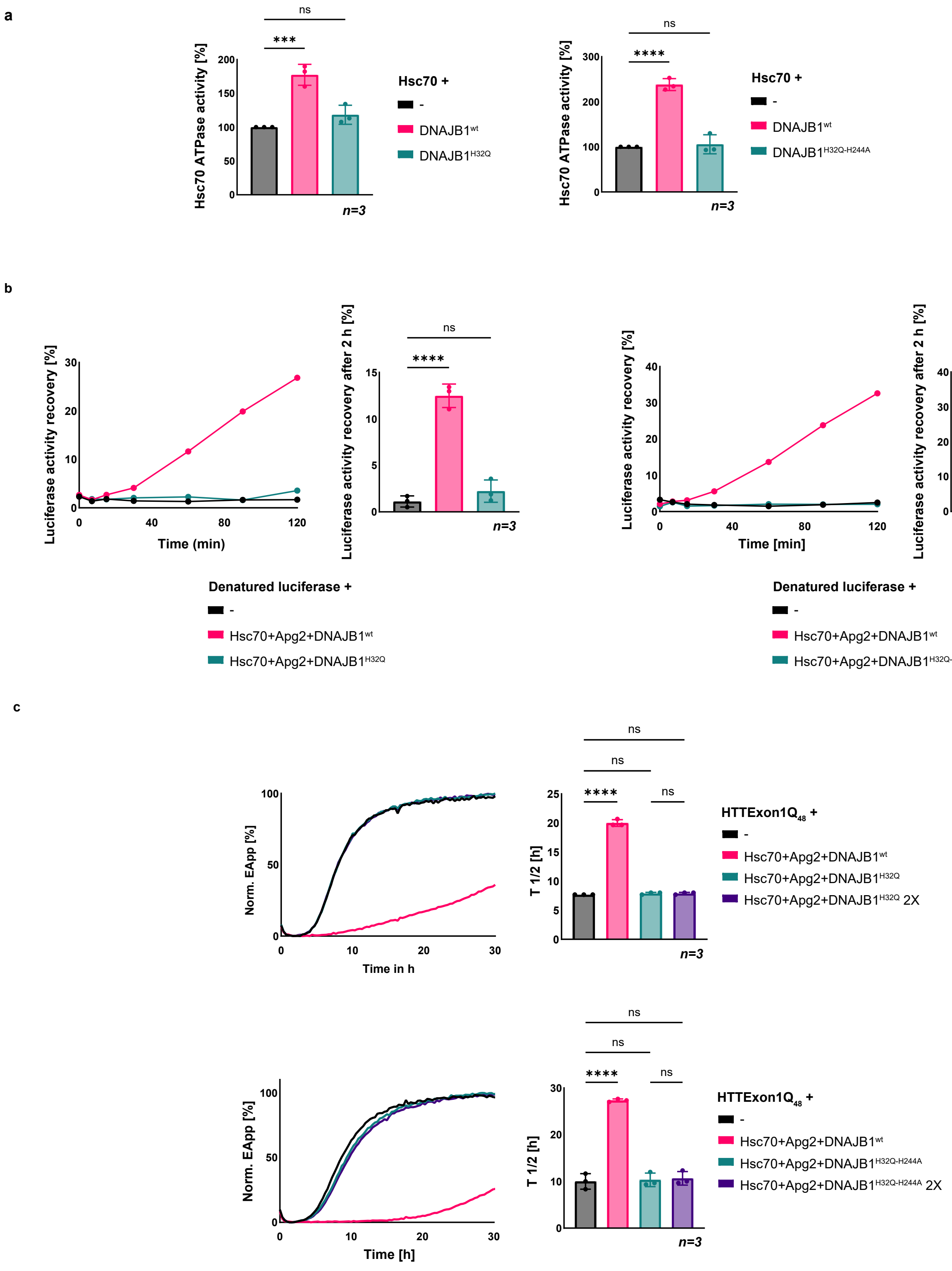
a
ns
***
Hsc70 ATPase activity [%]
200
150
100
50
0
n=3
Hsc70 +
-
DNAJB1wt
DNAJB1H32Q
ns
****
Hsc70 ATPase activity [%]
300
200
100
0
n=3
Hsc70 +
-
DNAJB1wt
DNAJB1H32Q-H244A
b
Luciferase activity recovery [%]
30
20
10
0
0
40
80
120
Time (min)
Luciferase activity recovery after 2 h [%]
ns
****
15
10
5
0
n=3
Luciferase activity recovery [%]
40
30
20
10
0
0
40
80
120
Time [min]
Luciferase activity recovery after 2 h [%]
ns
***
40
30
20
10
0
n=3
Denatured luciferase +
-
Hsc70+Apg2+DNAJB1wt
Hsc70+Apg2+DNAJB1H32Q
Denatured luciferase +
-
Hsc70+Apg2+DNAJB1wt
Hsc70+Apg2+DNAJB1H32Q-H244A
c
Norm. EApp [%]
100
50
0
0
10
20
30
Time in h
ns
ns
****
ns
T 1/2 [h]
25
20
15
10
5
0
n=3
HTTExon1Q48 +
-
Hsc70+Apg2+DNAJB1wt
Hsc70+Apg2+DNAJB1H32Q
Hsc70+Apg2+DNAJB1H32Q 2X
Norm. EApp [%]
100
50
0
0
10
20
30
Time [h]
ns
ns
****
ns
T 1/2 [h]
30
20
10
0
n=3
HTTExon1Q48 +
-
Hsc70+Apg2+DNAJB1wt
Hsc70+Apg2+DNAJB1H32Q-H244A
Hsc70+Apg2+DNAJB1H32Q-H244A 2X

**Supplementary figure 6. Analysis of the HPD motif of DNAJB1 in chaperone assays a)** ATPase activity of Hsc70 alone and upon addition of DNAJB1$^{H32Q}$ (left) or DNAJB1$^{H32Q\text{-}H244A}$ (right). All data were normalized to the intrinsic ATPase activity of Hsc70. One-way ANOVA analysis was used to evaluate the significance of the difference observed between Hsc70 alone and Hsc70 plus each mutant. Bars represent the mean value and error bars correspond to the mean SD. ****, p ≤ 0,0001; ***, p < 0,001; ns, not significant. **b)** Refolding of denatured luciferase by Hsc70, Apg2 and DNAJA1$^{H32Q}$ (top) or DNAJA1$^{H32Q\text{-}H244A}$ (bottom). A one-way ANOVA analysis of the amount of refolded luciferase after 2 h of incubation with chaperones is depicted on the right side of each graph. Bars represent the mean value and error bars correspond to the mean SD. ****, p < 0,0001; ***, *p* ≤ 0,001; *ns*, not significant. **c)** FRET assay of HTTExon1$Q_{48}$ aggregation and the effect of the addition of Hsc70, Apg2 and DNAJA1$^{H32Q}$ (top) or DNAJA1$^{H32Q\text{-}H244A}$ (bottom). The graph is a representative result of three independent experiments and a one-way ANOVA analysis of the half-life ($T_{1/2}$) of HTTExon1$Q_{48}$ aggregation under the tested conditions is depicted on the right. Error bars correspond to the SD. ****, *p* ≤ 0,0001; *ns*, not significant.

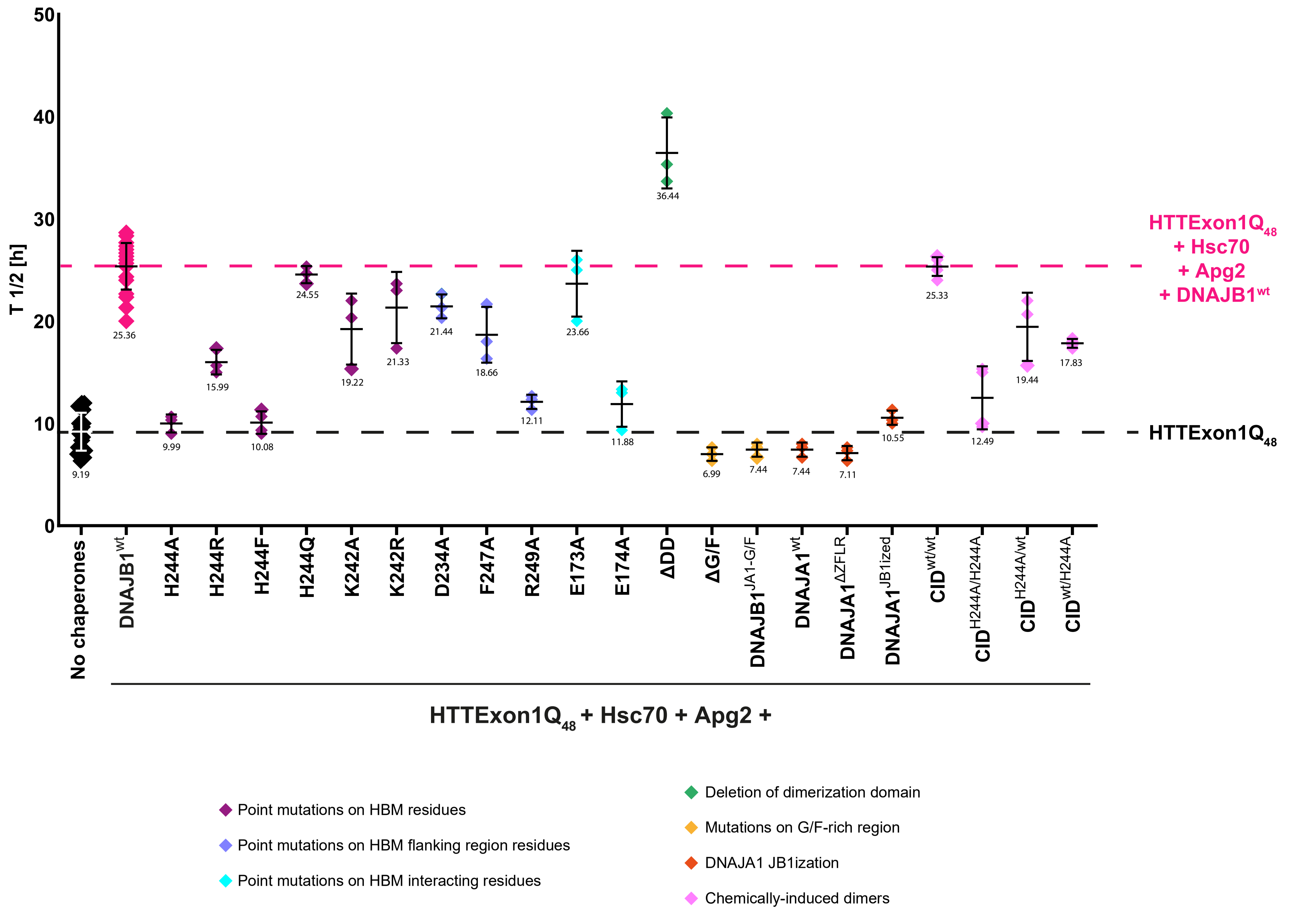

T 1/2 [h]
0
10
20
30
40
50
HTTExon1Q48 + Hsc70 + Apg2 + DNAJB1wt
HTTExon1Q48
9.19
25.36
9.99
15.99
10.08
24.55
19.22
21.33
21.44
18.66
12.11
23.66
11.88
36.44
6.99
7.44
7.44
7.11
10.55
25.33
12.49
19.44
17.83
No chaperones
DNAJB1wt
H244A
H244R
H244F
H244Q
K242A
K242R
D234A
F247A
R249A
E173A
E174A
ΔDD
ΔG/F
DNAJB1JA1-G/F
DNAJA1wt
DNAJA1ΔZFLR
DNAJA1JB1ized
CIDwt/wt
CIDH244A/H244A
CIDH244A/wt
CIDwt/H244A
HTTExon1Q48 + Hsc70 + Apg2 +
Point mutations on HBM residues
Point mutations on HBM flanking region residues
Point mutations on HBM interacting residues
Deletion of dimerization domain
Mutations on G/F-rich region
DNAJA1 JB1ization
Chemically-induced dimers

**Supplementary figure 7. Summary of the $T_{1/2}$ of HTTExon1$Q_{48}$ fibrilization** Depicted is the $T_{1/2}$ of HTTExon1$Q_{48}$ upon addition of Hsc70, Apg2 and each of the JDP described in this study (Figs 1, 2, 4, 5, 6, 7 and Supplementary Figure 5). The black dashed line corresponds to the mean $T_{1/2}$ of HTTExon1$Q_{48}$ fibrilization and the magenta dashed line corresponds to the $T_{1/2}$ of HTTExon1$Q_{48}$ fibrilization in the presence of Hsc70, Apg2 and DNAJB1$^{wt}$ and ATP regeneration system. Each diamond represents the $T_{1/2}$ of HTTExon1$Q_{48}$ fibrilization when DNAJB1$^{wt}$ is substituted by the variants shown on the x-axis. Each variant was tested in three independent experiments. The mean $T_{1/2}$ values (in h) are shown below each diamond. The error bars represent the mean SD. The color code of the diamonds indicates the type of variant/mutation used and is listed below the graph.
