# Supplementary material for: Identification of a HTT-specific binding motif in DNAJB1 essential for suppression and disaggregation of HTT
Source: Nat Commun. 2022 Aug 10;13:4692. doi: 10.1038/s41467-022-32370-5 (PMC9365803; doi:10.1038/s41467-022-32370-5)
Supplement: Supplementary file 3 — Reporting Summary [file 41467_2022_32370_MOESM3_ESM.pdf]

nature portfolio

Corresponding author(s): Janine Kirstein

Last updated by author(s): Jul 23, 2022

Reporting Summary

Nature Portfolio wishes to improve the reproducibility of the work that we publish. This form provides structure for consistency and transparency in reporting. For further information on Nature Portfolio policies, see our Editorial Policies and the Editorial Policy Checklist.

Statistics

For all statistical analyses, confirm that the following items are present in the figure legend, table legend, main text, or Methods section.

n/a	Confirmed	
	x	The exact sample size (*n*) for each experimental group/condition, given as a discrete number and unit of measurement
x		A statement on whether measurements were taken from distinct samples or whether the same sample was measured repeatedly
	x	The statistical test(s) used AND whether they are one- or two-sided<br>*Only common tests should be described solely by name; describe more complex techniques in the Methods section.*
	x	A description of all covariates tested
	x	A description of any assumptions or corrections, such as tests of normality and adjustment for multiple comparisons
	x	A full description of the statistical parameters including central tendency (e.g. means) or other basic estimates (e.g. regression coefficient) AND variation (e.g. standard deviation) or associated estimates of uncertainty (e.g. confidence intervals)
	x	For null hypothesis testing, the test statistic (e.g. *F*, *t*, *r*) with confidence intervals, effect sizes, degrees of freedom and *P* value noted<br>*Give P values as exact values whenever suitable.*
x		For Bayesian analysis, information on the choice of priors and Markov chain Monte Carlo settings
x		For hierarchical and complex designs, identification of the appropriate level for tests and full reporting of outcomes
x		Estimates of effect sizes (e.g. Cohen's *d*, Pearson's *r*), indicating how they were calculated

Our web collection on statistics for biologists contains articles on many of the points above.

Software and code

Policy information about availability of computer code

Data collection	Biochemical data was collected using the Tecan i-Control TM 1.10.1.0 software of the Infinite F200 PRO Tecan plate reader. Westernblot and filter retardation assays signals were detected using Image Studio TM software 4.0 from the LI-COR Odyssey Fc imaging system, CHARMM-GUI solvation builder, MD simulations: GROMACS 2018 version.
Data analysis	Raw data was treated in Microsoft's Excel and GraphPad Prism 8 and 9. Image data was treated in Fiji/Image J.Simulations were analyzed every 100 ps using the Visual Molecular Dynamics (VMD) software, the open-source community-developed PLUMED library version 2.6 and plots visualized by Python version 3.7. Crosslinking analysis was performed using XlinkX. CD analysis was done with Pro-Data Chirascan v.4.2.22. Clustal omega was used for multiple sequence alignments and BoxShade (discontinued server) was used for their formatting. WebLogo 2.8.2 was used for amino acids conservation analysis. Maestro Software Suite Version 12.7 was used to build protein homology models.

For manuscripts utilizing custom algorithms or software that are central to the research but not yet described in published literature, software must be made available to editors and reviewers. We strongly encourage code deposition in a community repository (e.g. GitHub). See the Nature Portfolio guidelines for submitting code & software for further information.

Data

Policy information about availability of data

All manuscripts must include a data availability statement. This statement should provide the following information, where applicable:

- Accession codes, unique identifiers, or web links for publicly available datasets
- A description of any restrictions on data availability
- For clinical datasets or third party data, please ensure that the statement adheres to our policy

The in silico data of this study like structure and trajectory files are made available under (https://doi.org/10.5281/zenodo.6365426).
PLUMED input files required to reproduce the results reported in this study are available on PLUMED-NEST (www.plumed-nest.org), the public repository of the PLUMED consortium as plumID: 22.012.
The mass spectrometry proteomics data have been deposited to the ProteomeXchange Consortium via the PRIDE partner repository (http://www.proteomexchange.org) with the dataset identifier PXD031214.
Protein structures from the PDB shown used in this study are: 3AGZ [http://doi.org/10.2210/pdb3AGZ/pdb], 3C7N [http://doi.org/ 10.2210/pdb3C7N/pdb], and 1NLT [http://doi.org/ 10.2210/pdb1NLT/pdb].

Human research participants

Policy information about studies involving human research participants and Sex and Gender in Research.

Reporting on sex and gender	N/A
Population characteristics	N/A
Recruitment	N/A
Ethics oversight	N/A

Note that full information on the approval of the study protocol must also be provided in the manuscript.

Field-specific reporting

Please select the one below that is the best fit for your research. If you are not sure, read the appropriate sections before making your selection.

- [x] Life sciences
- [ ] Behavioural & social sciences
- [ ] Ecological, evolutionary & environmental sciences

For a reference copy of the document with all sections, see nature.com/documents/nr-reporting-summary-flat.pdf

Life sciences study design

All studies must disclose on these points even when the disclosure is negative.

Sample size	Only applicable to the cell culture data (Fig. 9) were we have analyzed between 700-800 individual cells for each condition to ensure robustness of the acquired data. Sample sizes was determined based on pilots experiments standard sample sizes used in this scientific community.
Data exclusions	No data were excluded.
Replication	All of the results shown in this manuscript result from a minimum of three independent experiments. Additionally, we have observed reproducibility of our results when experiments are performed by different experimenters in different lab locations and using proteins from independently-purified batches. We confirm that our experimental replicates were reproducible and successful.
Randomization	Samples were allocated into random experimental groups. In addition, the different experimental groups were repeated on different instruments with different batches of purified proteins.
Blinding	The investigators were blinded for the experimental or analysis step.

Reporting for specific materials, systems and methods

We require information from authors about some types of materials, experimental systems and methods used in many studies. Here, indicate whether each material, system or method listed is relevant to your study. If you are not sure if a list item applies to your research, read the appropriate section before selecting a response.

Materials & experimental systems

n/a	Involved in the study
☐	☒ Antibodies
☐	☒ Eukaryotic cell lines
☒	☐ Palaeontology and archaeology
☒	☐ Animals and other organisms
☒	☐ Clinical data
☒	☐ Dual use research of concern

Methods

n/a	Involved in the study
☒	☐ ChIP-seq
☒	☐ Flow cytometry
☒	☐ MRI-based neuroimaging

Antibodies

Antibodies used	Anti-DNAJB1 from Proteintech (cat.no. 13174-AP) and anti-EGFP from Enzo (ENZ-ABS141-0200), Anti-actin antibody from Sigma (#A5441); Anti-mouse-HRP from ThermoFisher (#31430), Anti-rabbit IRDue 680 from LI-COR Biosciences.
Validation	All antibodies were validated for KD and KO by the producers. It targets the antigen in WB, IP, IF, FC applications and detect human proteins:<br>anti-DNAJB1 from Proteintech: https://www.ptglab.com/products/DNAJB1-Antibody-13174-1-AP.htm<br>anti-GFP antibody from Enzo: https://www.enzolifesciences.com/ENZ-ABS141/green-fluorescent-protein-monoclonal-antibody-b34/<br>anti-actin antibody from Sigma: https://www.sigmaaldrich.com/DE/de/product/sigma/a5441<br>Anti-mouse-HRP from ThermoFisher: https://www.thermofisher.com/antibody/product/Goat-anti-Mouse-IgG-H-L-Secondary-Antibody-Polyclonal/31430

Eukaryotic cell lines

Policy information about cell lines and Sex and Gender in Research

Cell line source(s)	The source of HEK293 cells is the DSMZ (German Collection of Microorganisms and Cell Cultures). This information is also available in the manuscript.
Authentication	Cells were not authenticated by us.
Mycoplasma contamination	Cells were not tested for mycoplasma contamination by us, but by the cell culture labs of the FMP (Leibniz Institute for Molecular Pharmacology) Berlin.
Commonly misidentified lines (See ICLAC register)	No commonly misidentified cell lines were used in our study.
