# Supplementary material for: Identification of a HTT-specific binding motif in DNAJB1 essential for suppression and disaggregation of HTT
Source: Nat Commun. 2022 Aug 10;13:4692. doi: 10.1038/s41467-022-32370-5 (PMC9365803; doi:10.1038/s41467-022-32370-5)
Supplement: Supplementary file 4 — Source data [file 41467_2022_32370_MOESM4_ESM.zip › Source data/Uncropped gels and blots_Nature Comms_Ayala Mariscal_final.pdf]

Uncropped gels and blots

Fig. 8b

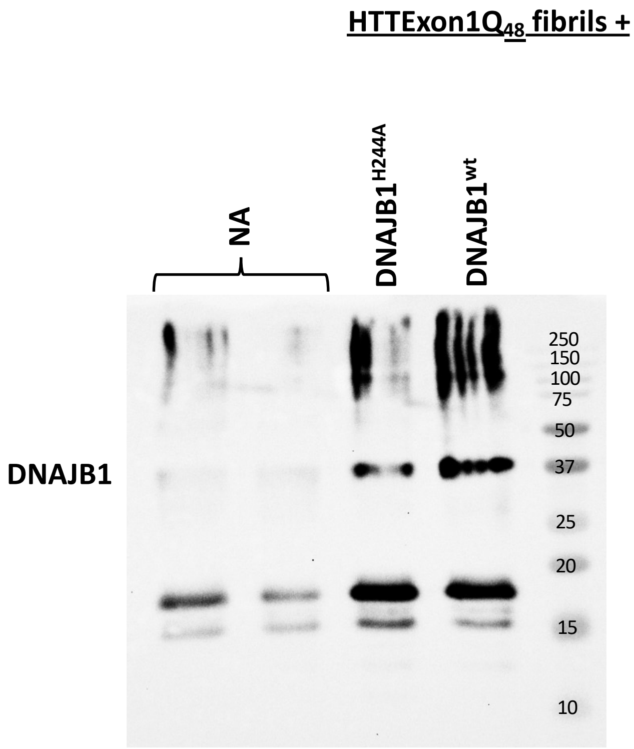

NA: not assessed/not shown in figures

Fig. 9a

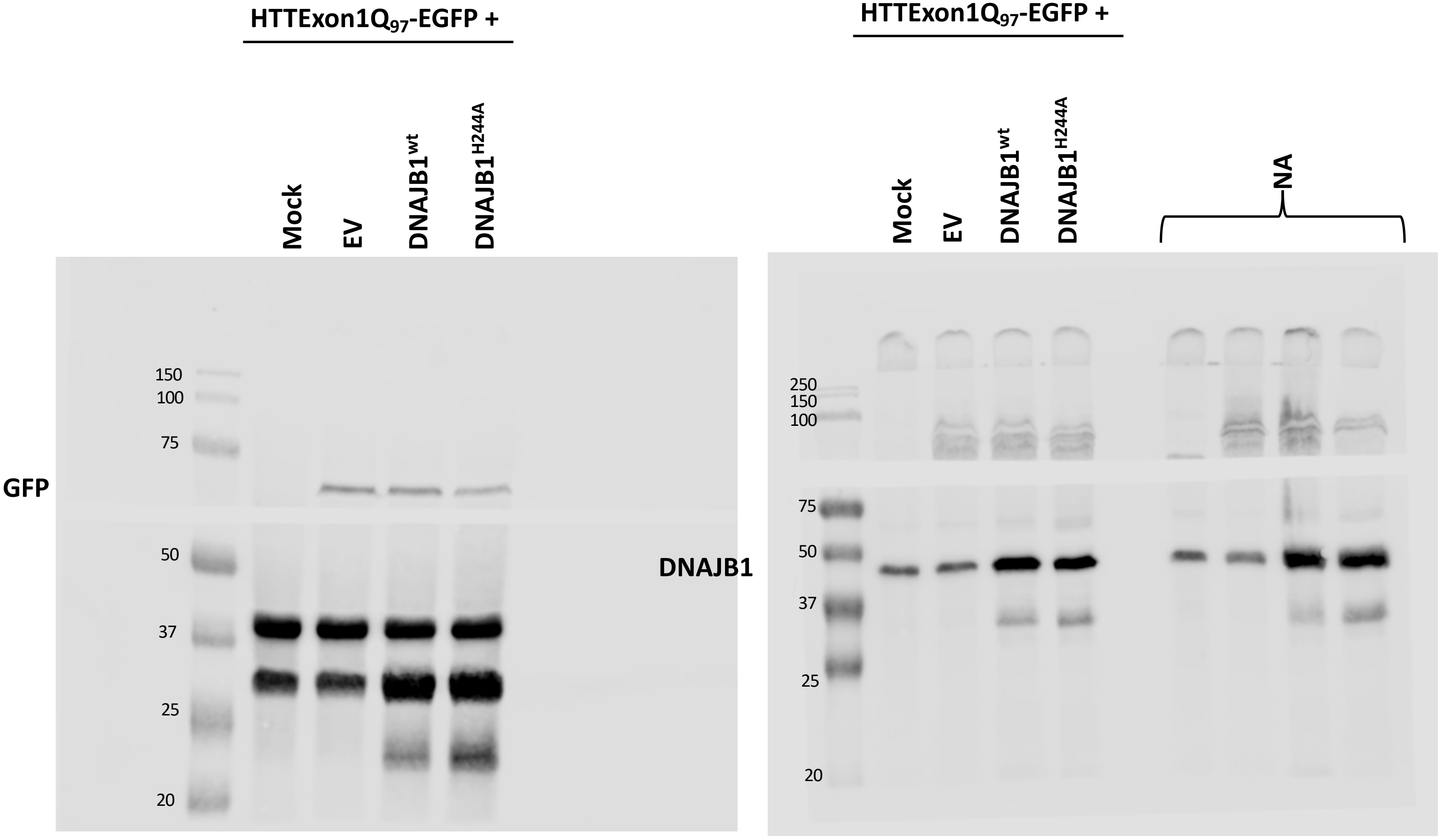

NA: not assessed/not shown in figures

Fig. 9a

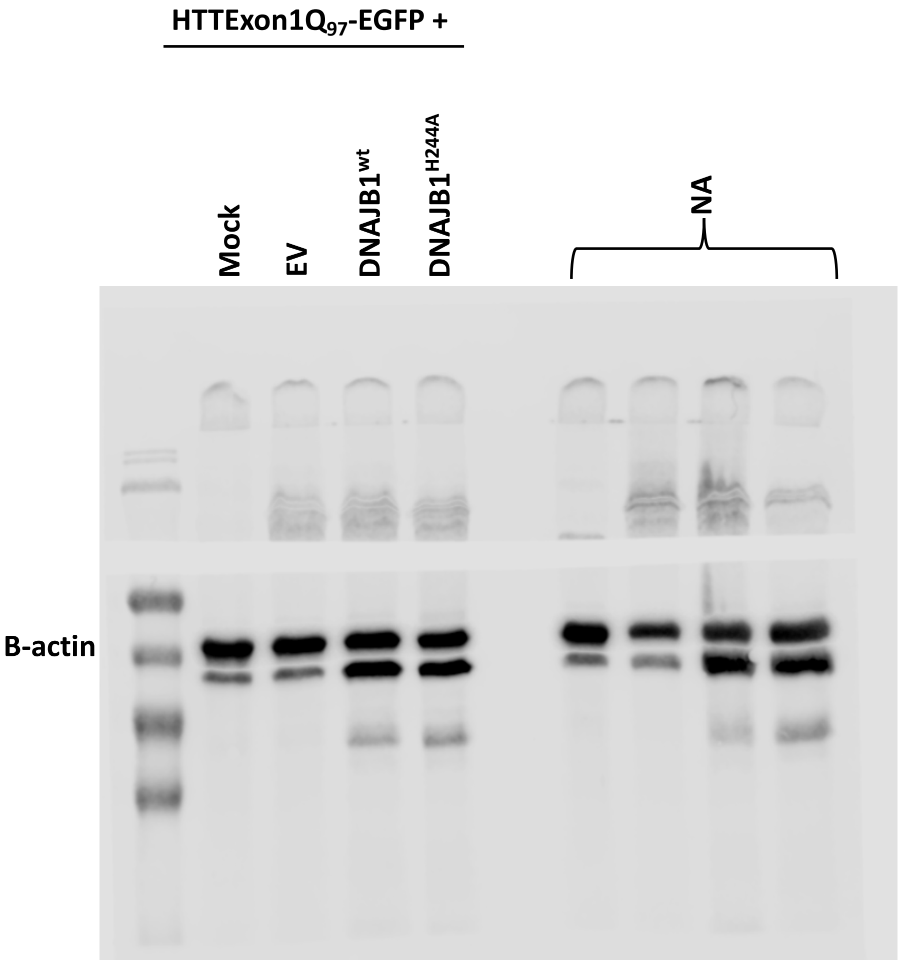

Fig. 9b

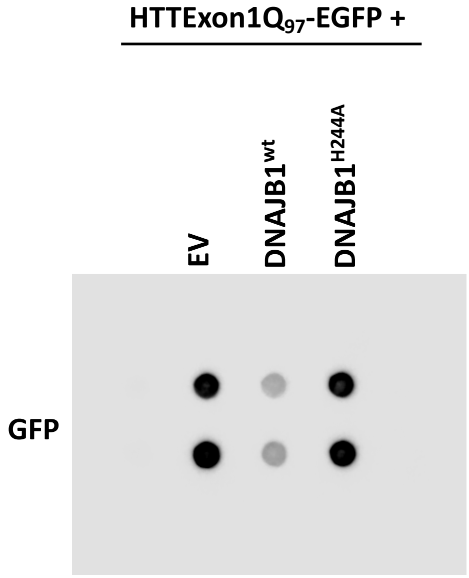

NA: not assessed/not shown in figures

Fig. 9e

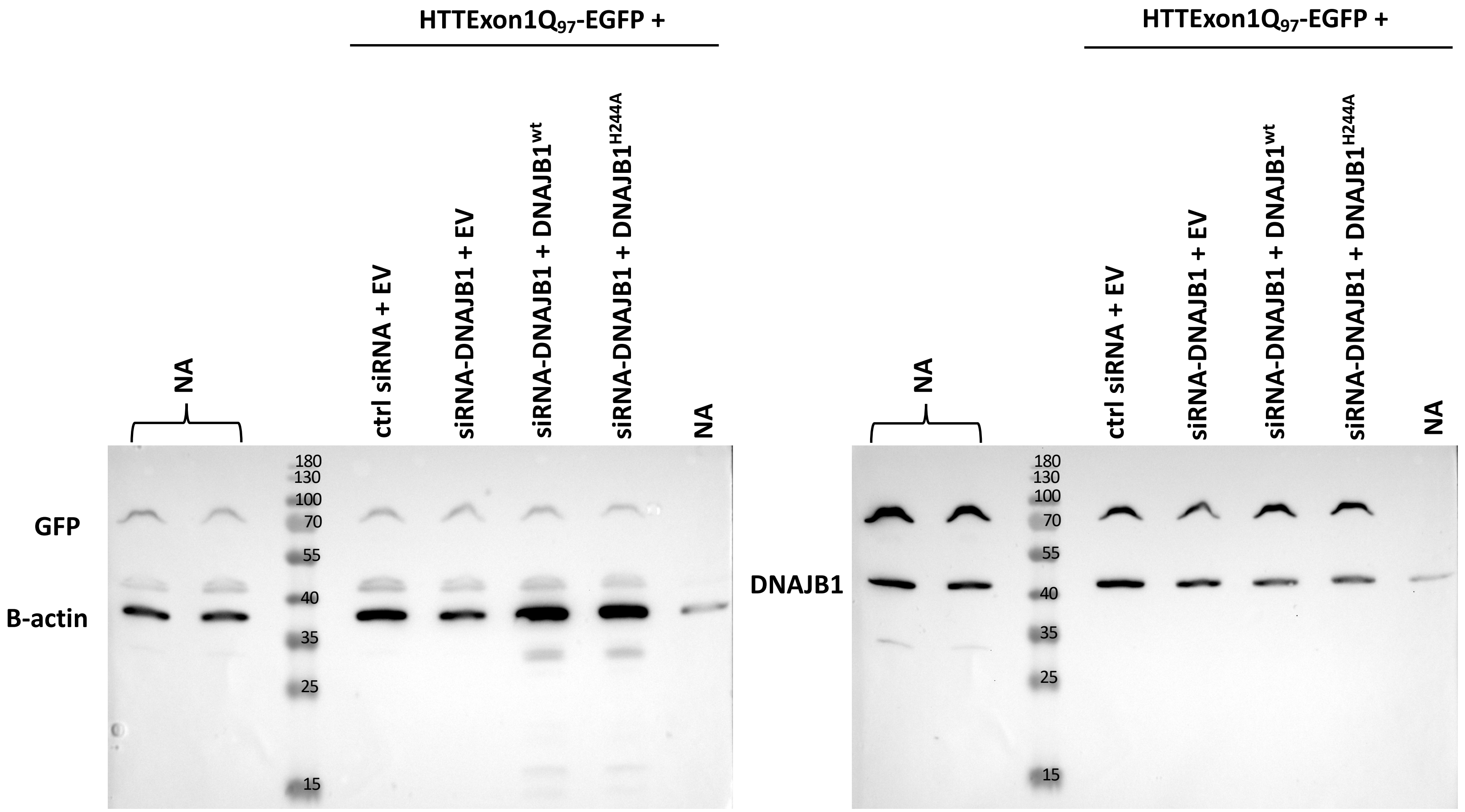
HTTExon1Q97-EGFP +
NA
ctrl siRNA + EV
siRNA-DNAJB1 + EV
siRNA-DNAJB1 + DNAJB1wt
siRNA-DNAJB1 + DNAJB1H244A
NA
GFP
B-actin
180
130
100
70
55
40
35
25
15
HTTExon1Q97-EGFP +
NA
ctrl siRNA + EV
siRNA-DNAJB1 + EV
siRNA-DNAJB1 + DNAJB1wt
siRNA-DNAJB1 + DNAJB1H244A
NA
DNAJB1
180
130
100
70
55
40
35
25
15

Supplementary Figure 2a

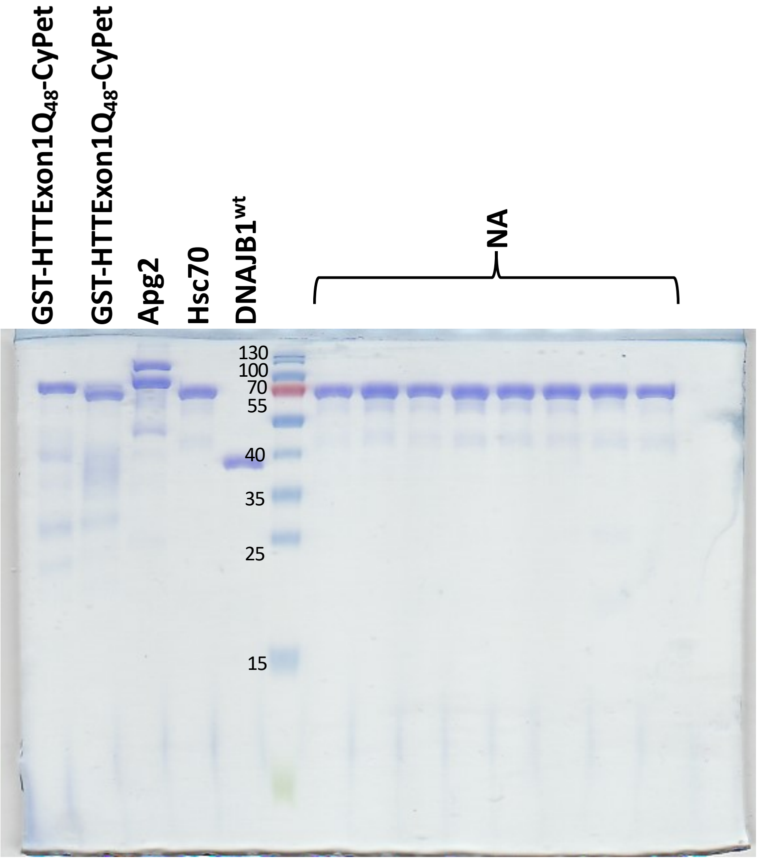

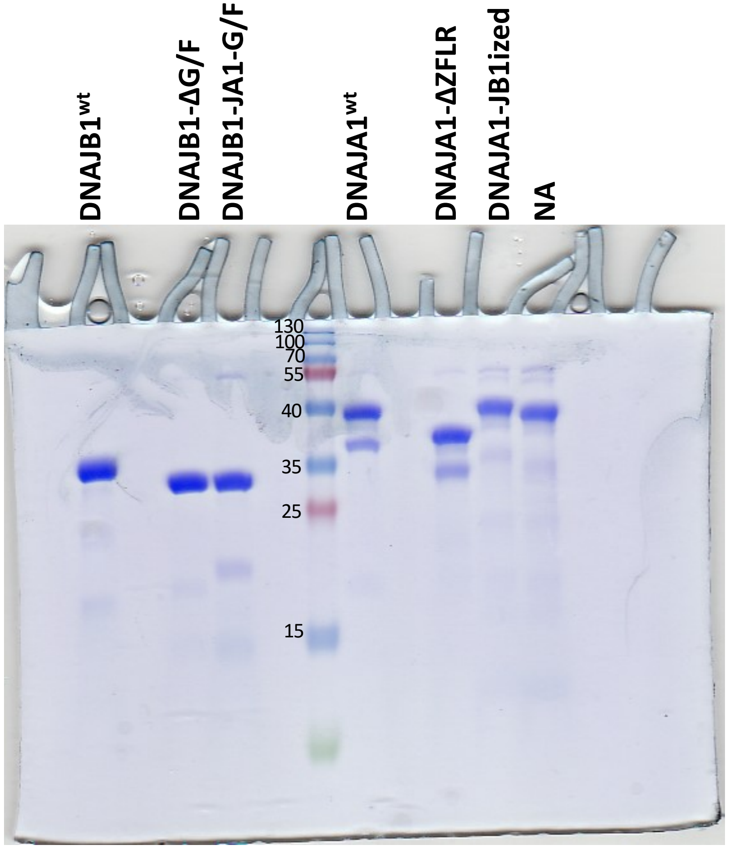

NA: not assessed/not shown in figures

Supplementary Figure 2a

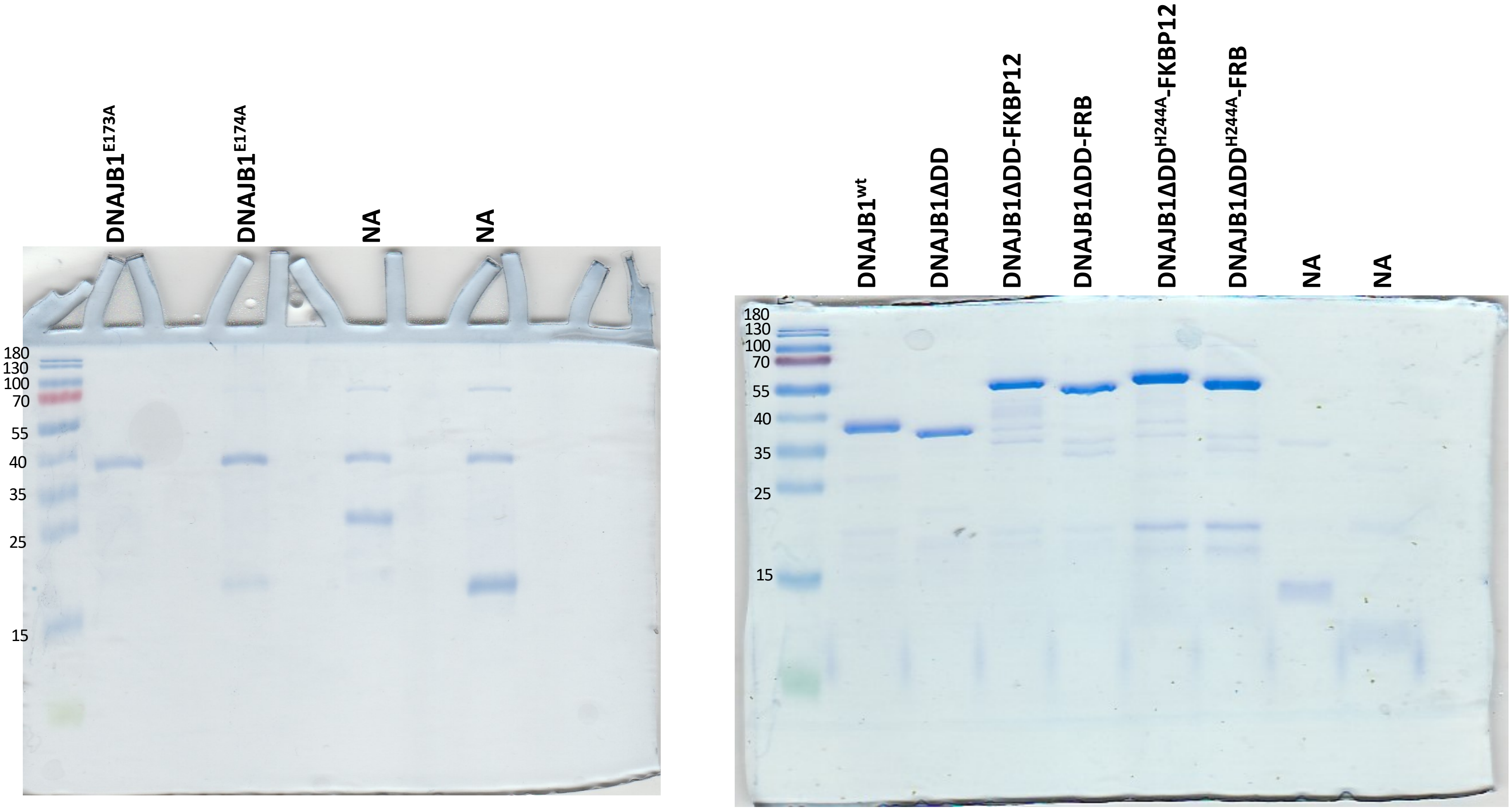

NA: not assessed/not shown in figures

Supplementary Figure 2a

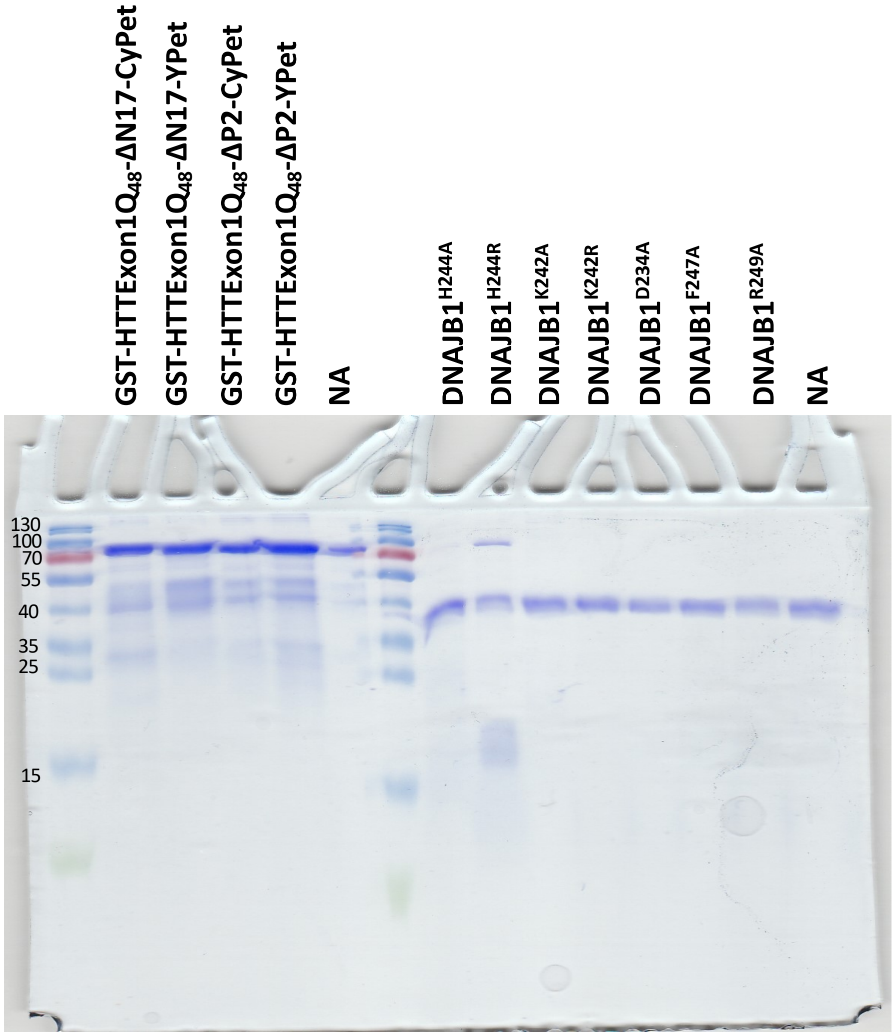

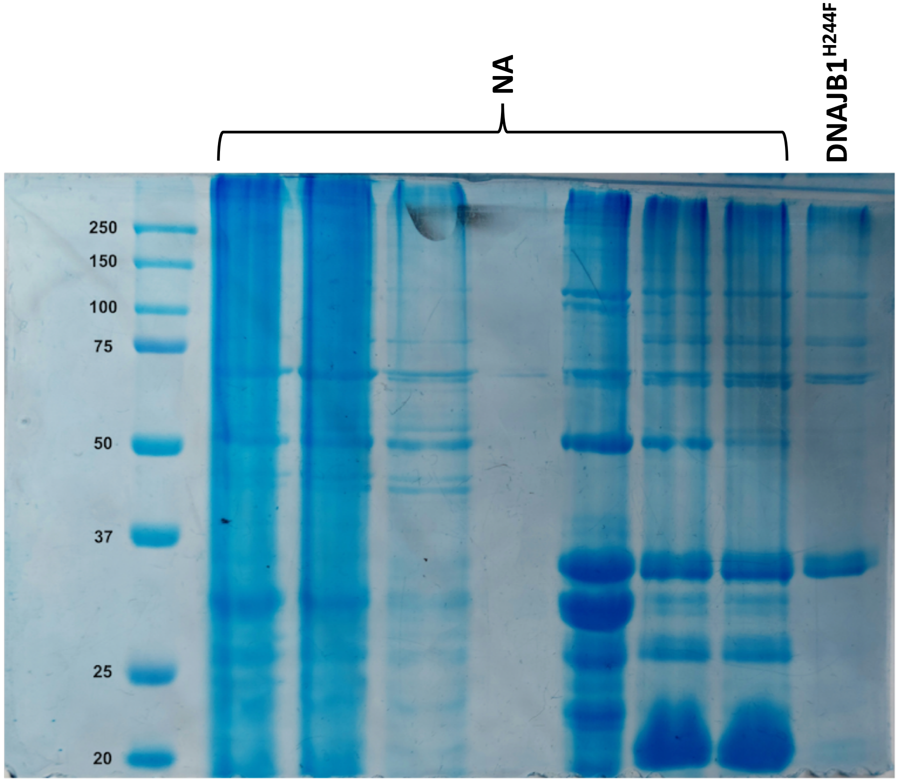

NA: not assessed/not shown in figures

Supplementary Figure 2a

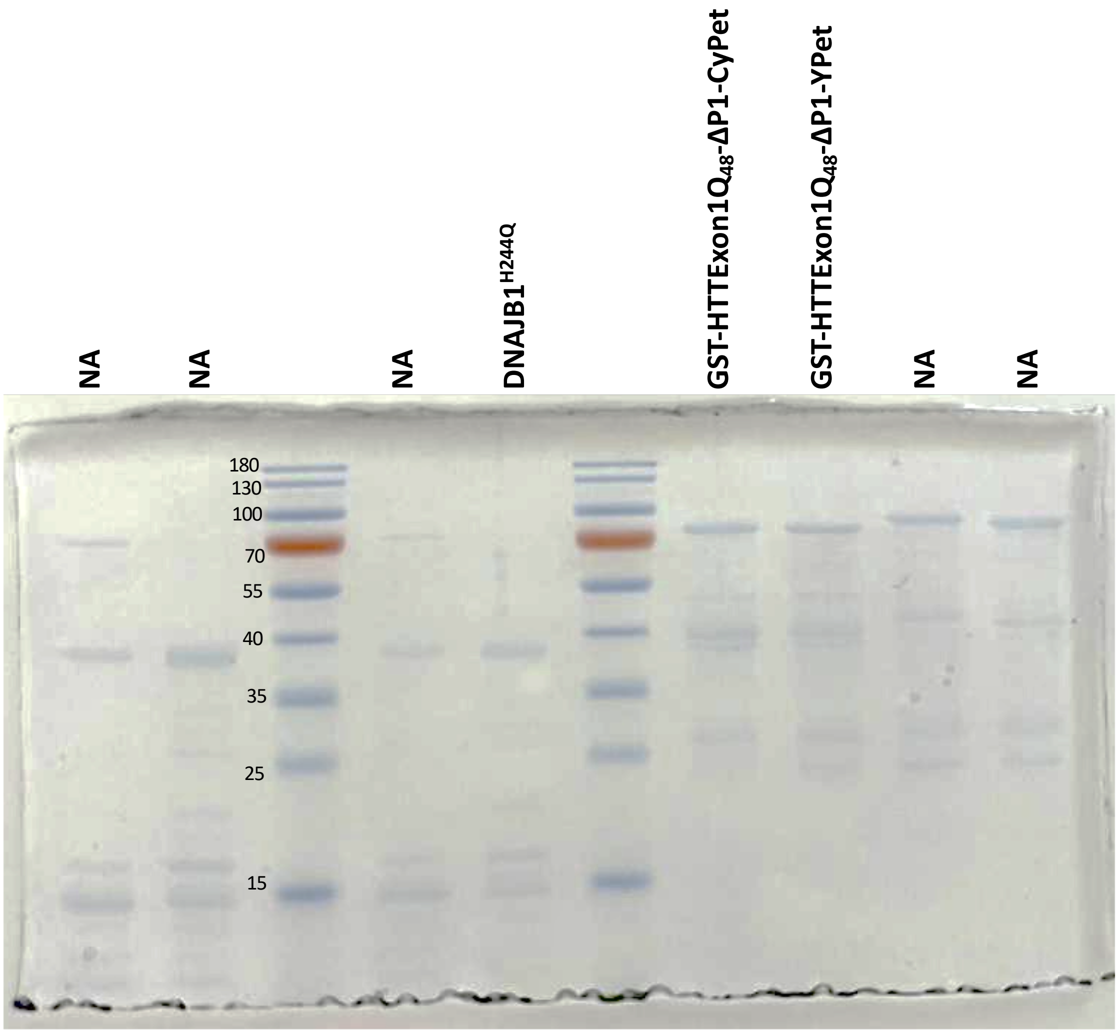

NA: not assessed/not shown in figures
